# Endothelial TGFβ signaling modulates choroidal neovascularization severity via myeloid–endothelial cell interaction

**DOI:** 10.3389/fimmu.2026.1813788

**Published:** 2026-06-26

**Authors:** Anja Schlecht, Lisa Müllerbauer, Katja Fitz, Bianka Brunne, Nico Hofmann, Christian Müller, Jost Hillenkamp, Süleyman Ergün, Andreas Neueder, Barbara M. Braunger

**Affiliations:** 1 Institute of Neuroanatomy, University Medical Center Hamburg-Eppendorf, Hamburg, Germany; 2 Institute of Anatomy and Cell Biology, Julius-Maximilians-University Wuerzburg, Wuerzburg, Germany; 3 Bioinformatics Core, University Medical Center Hamburg-Eppendorf, Hamburg, Germany; 4 Department of Ophthalmology, Universitaetsklinikum Wuerzburg, Wuerzburg, Germany; 5 Atlas University Research Center (ARC), Atlas University, Istanbul, Türkiye; 6 Institute for Molecular Neurogenetics, Center for Molecular Neurobiology (ZMNH), University Medical Center Hamburg-Eppendorf, Hamburg, Germany

**Keywords:** age-related macular degeneration, blood retinal barrier, choroidal neovascularization, endothelial cells, microglia, mononuclear phagocytes, TGFβ signaling

## Abstract

**Introduction:**

The blood-retinal barrier (BRB) is essential for maintaining retinal homeostasis, consequently its disruption contributes to pathological angiogenesis in diseases such as neovascular age-related macular degeneration (nAMD). Choroidal neovascularization (CNV) is considered as a hallmark of nAMD. These newly formed vessels break through the BRB leading to rapid and severe vision loss. Here, we investigated how endothelial TGFβ signaling interacts with mononuclear phagocytes (MP), such as microglia to regulate choroidal neovascularization (CNV).

**Methods:**

In this study, we used a laser-induced CNV model in mice with endothelial-specific Tgfbr2 deletion and concomitant MP depletion via PLX5622.

**Results:**

We demonstrate that loss of endothelial TGFβ signaling significantly exacerbates CNV. Strikingly, this effect is fully rescued by MP depletion. Transcriptome and RNA localization of CNV lesions identified fibrinogen alpha chain (*Fga*) as a MP-derived factor that is exclusively upregulated in mice without endothelial TGFβ signaling.

**Discussion:**

These findings suggest a novel TGFβ-dependent interaction between endothelial cells and MP that promotes angiogenesis through microglia derived *Fga* expression.

## Introduction

The retina, as an extension of the brain, is part of the central nervous system (1). Due to its high metabolic activity, the retina needs a sufficient blood supply and a tightly regulated microenvironment to maintain proper neuronal function (1, 2). The blood-retinal barriers (BRBs) separate the retina from the systemic circulation, thereby preserving the microenvironment and regulating molecular exchange between the blood and neuronal tissue (3, 4). There are two physiological barriers: the inner (iBRB) and the outer (oBRB) blood retinal barrier (5, 6). The iBRB is formed by tight junctions between endothelial cells (6, 7), but its integrity relies on the contribution of other cell types such as mural cells (comprising vascular smooth muscle cells and pericytes), glial cells and neurons that collectively form the neurovascular unit (NVU) (8).

Endothelial cells share their basement membrane with pericytes, which provide structural stability and regulatory input (1, 8, 9), while glial cells such as astrocytes, Müller cells, and microglia actively modulate barrier properties and vascular function (10–13). Structurally and functionally, the retinal NVU closely parallels the NVU of the blood brain barrier (1, 8).

The oBRB is established by tight junctions between adjacent retinal pigment epithelial (RPE) cells (6, 14, 15). The RPE forms a monolayer of pigmented epithelial cells that separates the neural retina from the underlying fenestrated endothelium of the choriocapillaris, with Bruch’s membrane interposed between them, thereby regulating the extraction of nutrients from the blood and their delivery to photoreceptors (7, 15). Cells of the RPE also clear metabolic waste, and the close relationship between the RPE and photoreceptor layer is essential of visual function (7, 15). Disruption of oBRB integrity is strongly implicated in retinal diseases including age-related macular degeneration (AMD) (7).

The neovascular form of AMD (nAMD) is characterized by the development of choroidal neovascularization (CNV), which leads to rapid, severe and irreversible vision loss (16–18). CNV originate from the choroidal vasculature, penetrate Bruch’s membrane, and subsequently breach the oBRB to grow into the subretinal space, often accompanied by edema formation and hemorrhage (19, 20). Vascular endothelial growth factor (VEGF), which stimulates endothelial cell proliferation is a potent driver of CNV formation (21). Anti-VEGF therapy has substantially improved nAMD treatment, however, its efficacy is limited by the short intraocular half-life of antibodies, necessitating repeated intravitreal injections (22). Moreover, approximately one-third of nAMD patients lose vision despite continuous anti-VEGF therapy (22, 23), suggesting the involvement of additional molecular mediators and/or cell populations in disease progression of nAMD.

In a previous study we found that the deletion of endothelial transforming growth factor β (TGFβ) signaling during ocular development results in spontaneous CNV formation (24), indicating a role for TGFβ signaling in nAMD pathogenesis. TGFβ signaling is initiated by binding of TGFβ1, TGFβ2, or TGFβ3, to TGFβ receptor II (TGFβRII), which subsequently forms a complex with TGFβRI to activate SMAD2/3–SMAD4 signaling and regulate TGFβ-dependent gene expression (25, 26). Beyond its broad regulatory functions (27, 28) TGFβ signaling plays a crucial role in maintaining the immune privilege in the eye (29–31).

Consistent with this, we observed accumulation of mononuclear phagocytes (MP), such as resident microglia, at CNV lesions in mice with a deletion of TGFβ signaling in endothelial cells (24). Notably, microglia in particular are increasingly being linked to the pathological process of CNV formation (32, 33).

To investigate whether MP interact with endothelial TGFβ signaling to promote CNV formation, we inhibited endothelial TGFβ signaling concomitant with MP depletion using PLX5622, a colony stimulating factor 1 receptor (CSF1R) inhibitor, in the background of laser-induced CNVs. This widely used experimental CNV model enables to study molecular and cellular mechanisms underlying CNV by triggering pathological angiogenesis through targeted disruption of Bruch’s membrane and the oBRB (34).

Using this approach, we found that deletion of endothelial TGFβ signaling in the presence of MP led to significantly enlarged CNV lesions, a phenotype rescued by MP depletion. RNA sequencing of CNV lesions identified fibrinogen alpha chain (*Fga*) as potential mediator of this interaction, a finding that was validated by RNAscope *in situ* hybridization and immunohistochemistry.

## Results

### Deletion of endothelial TGFβ signaling exacerbates CNV formation

**Figure 1:**
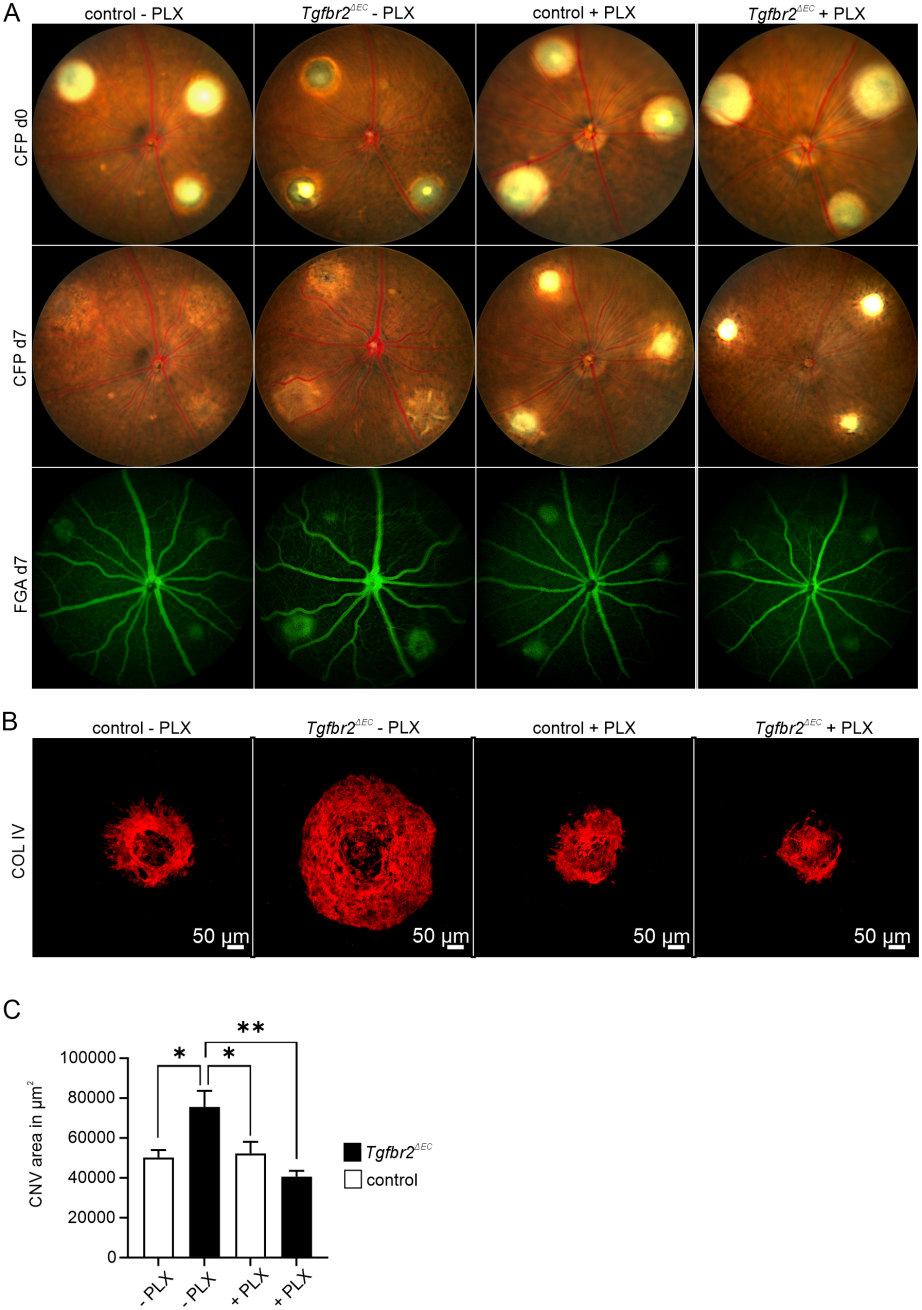
Deletion of endothelial TGFβ signaling acts proangiogenic and exacerbates CNV formation. **(A)** *In vivo* imaging of CNV lesions at day 0 (d0) and day seven (d7) after laser treatment. Color fundus photography (CFP) and fluorescein angiography (FLA) were used to visualize CNV formation in control and *Tgfbr2^ΔEC^* mice each group with and without PLX5622 treatment. **(B)** Representative *ex vivo* immunohistochemical staining for COLIV in choroidal flatmounts of control and *Tgfbr2^ΔEC^* mice each group with and without PLX5622 treatment. **(C)** Quantification of CNV lesion size based on COLIV staining as shown in **(B)** (control – PLX n = 5; *Tgfbr2^ΔEC^* – PLX n = 6; control + PLX n = 6; *Tgfbr2^ΔEC^* + PLX n = 6). Data are mean ± SEM, two-way ANOVA; interaction endothelial TGFβ signaling and the presence of microglia on lesion size *p* = 0.0051, * adj. p ≤ 0.5, ** adj. *p* ≤ 0.01.

**Figure 2:**
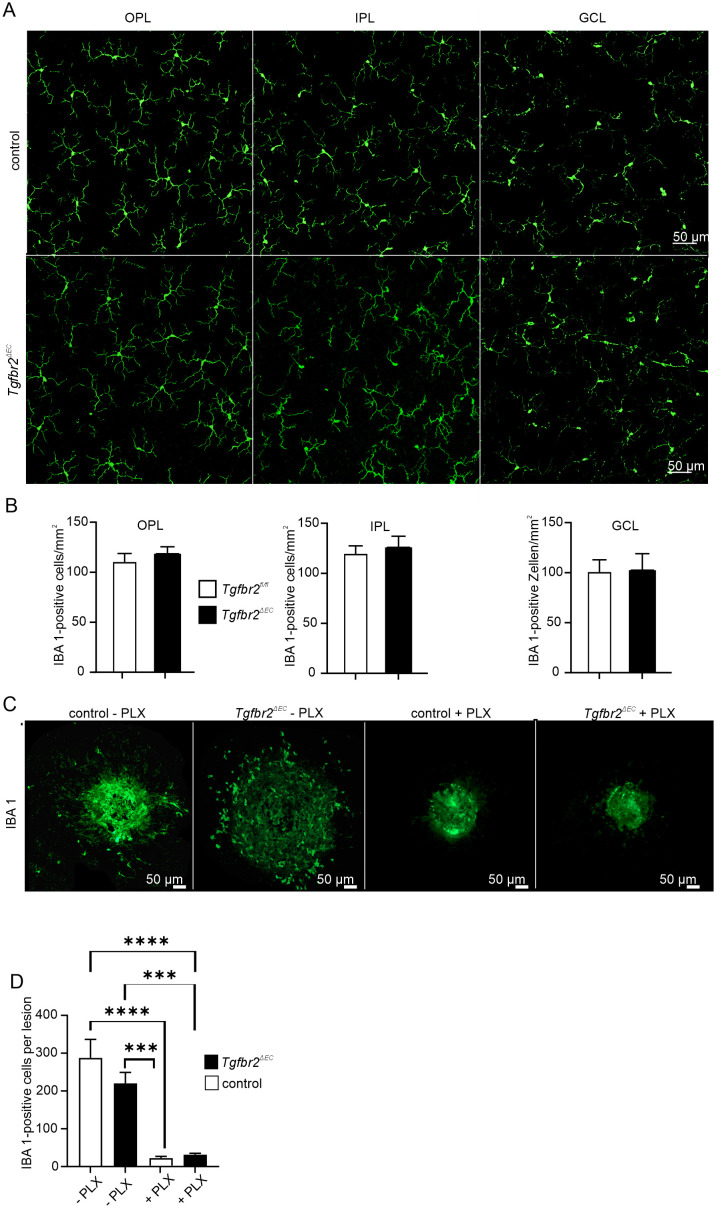
Deletion of endothelial TGFβ signaling does not change microglia cell density. **(A)** Immunohistochemical staining of retinal flatmounts against IBA-1. **(B)** Quantification of microglia cell numbers without laser-induced CNV in the retinal ganglion cell layer (GCL), the inner plexiform layer (IPL) and the outer plexiform layer (OPL) of control (n = 5) and in *Tgfbr2^ΔEC^* (n = 3) mice. **(C, D)** Immunohistochemical staining against IBA-1 **(C)** and quantification **(D)** of accumulating mononuclear phagocyte cell numbers seven days following laser-induced CNV in control and in *Tgfbr2^ΔEC^* mice with and without PLX5622 treatment (control – PLX n = 5; *Tgfbr2^ΔEC^* – PLX n = 6; control + PLX n = 6; *Tgfbr2^ΔEC^* PLX n = 6). Data are mean ± SEM, two-way ANOVA, *** adj. p ≤ 0.001, **** adj. *p* ≤ 0.0001.

To study the effect of endothelial TGFβ signaling on the formation and severity of choroidal neovascularization (CNV), CNVs were induced in mice with an endothelial deletion of TGFβRII (referred to as: *Tgfbr2^ΔEC^*) and TGFβRII competent (referred to as: controls) littermates using a laser to break Bruch’s membrane and the oBRB (**Figure 1**). Successful deletion of TGFβRII in the choroidal and retinal endothelium in *Tgfbr2^ΔEC^* mice is shown in **Supplementary Figure 1**. Directly after laser treatment, color fundus photography (CFP) showed no morphological or size-specific differences between the laser foci in *Tgfbr2^ΔEC^* mice compared to control littermates (**Figure 1A**). Yet, after seven days, the fluorescein *in vivo* angiography (FLA) revealed visibly enlarged CNV lesions in *Tgfbr2^ΔEC^* mice (**Figure 1A**). We quantified the area of the laser-induced CNVs using immunohistochemical staining against Collagen IV (COL IV) on choroidal flatmounts, and observed a significant increase in CNV lesion size in *Tgfbr2^ΔEC^* mice compared to control littermates (control - PLX: 50242 ± 3744, n=5; *Tgfbr2^ΔEC^* - PLX: 75088 ± 8125; n=6, *p* = 0.0328; **Figures 1B, C**). To determine whether MP contribute to the increased CNV development in *Tgfbr2^ΔEC^* mice, we depleted MP using PLX5622 in a subset of control and *Tgfbr2^ΔEC^* mice. This resulted in a robust and significant depletion of microglia in the ganglion cell layer (GCL), inner plexiform layer (IPL) and outer plexiform layer (OPL) of the central and peripheral retina (**Supplementary Figure 2**) and of MP located at CNV lesions (**Figure 2D**). Both, *in vivo* FLA images as well as quantification of CNV area on choroidal flatmounts demonstrated that MP depletion in *Tgfbr2^ΔEC^* mice reduced CNV lesion size to the level observed in TGFβRII competent control animals (*Tgfbr2^ΔEC^* + PLX = 40637µm² ± 2877, n=6, *p* = 0.0017; **Figure 1**). In this context, two-way ANOVA analyses revealed a significant interaction between endothelial TGFβ signaling and the presence of MP on lesion size. However, when comparing the two TGFβRII competent control groups with (+ PLX) and without (- PLX) MP depletion, we detected no difference in CNV lesion size (control - PLX = 50242µm² ± 3744, control + PLX = 51822 µm² ± 5788; **Figure 1**). Taken together, these data show that endothelial-specific loss of TGFβ signaling promotes angiogenesis resulting in significantly larger CNV lesions. Pharmacological MP depletion though PLX treatment completely reversed this proangiogenic phenotype in *Tgfbr2^ΔEC^* mice, while having no measurable impact on CNV lesion size in TGFβRII competent control animals. Thus, these results strongly suggest a direct proangiogenic interaction between endothelial TGFβ signaling and mononuclear phagocytes, such as microglia cells.

### Deletion of endothelial TGFβ signaling does not affect microglia cell numbers

To investigate, whether endothelial TGFβ signaling affects the number of MP, which will most likely represent microglia cells in the otherwise healthy retina and in turn might contribute to the pro-angiogenic phenotype observed in *Tgfbr2^ΔEC^*, we quantified IBA1-positive MP in the retinae of control and *Tgfbr2^ΔEC^* mice without laser-induced CNV, but found no significant differences (control n = 5; *Tgfbr2^ΔEC^* n = 3; GCL: control = 100.6 ± 12.28, *Tgfbr2^ΔEC^* = 103.3 ± 15.71; IPL: control = 119.4 ± 7.959, *Tgfbr2^ΔEC^* = 126.7 ± 10.36; OPL: control = 110.2 ± 8.555, *Tgfbr2^ΔEC^* = 119.2 ± 6.276; **Figures 2A, B**). We therefore conclude that deletion of endothelial TGF-β signaling does not, in itself, affect the number of MP cells, such as microglia, in an otherwise healthy retina.

Yet as briefly outlined before, following PLX5622 treatment, we detected a massive and significant reduction in MP accumulation in both, control and *Tgfbr2^ΔEC^* retinae (**Supplementary Figure 2C**; peripheral and central retina) and CNV lesions (mean ± SEM; control - PLX = 287.4 ± 49.12, n = 5; control + PLX = 19.69 ± 4.772, n = 6, adj. *p* < 0.0001; *Tgfbr2^ΔEC^* - PLX = 220.1 ± 29.1, n = 6; *Tgfbr2^ΔEC^* + PLX = 31.35 ± 3.950, n = 6, adj. *p* = 0.0003; **Figures 2C, D**) with no significant difference in MP numbers between the two genotypes.

### Transcriptional profiling of CNV lesions in *Tgfbr2^ΔEC^* mice and controls identifies *Fga* as a possible regulator for CNV development

Since our data indicate that loss of endothelial TGFβ signaling permits mononuclear phagocytes to regulate or express molecular factors that promote angiogenesis and thereby increase CNV severity in *Tgfbr2^ΔEC^* mice, we next assessed the functional contribution of MP cells to this phenotype. To identify molecular mediators underlying this process, we performed RNA sequencing of CNV-lesioned choroids from all four experimental groups. To enrich for putative MP-derived factors regulated by endothelial TGFβ signaling, we filtered for protein-coding genes that were differentially expressed (adj. *p* ≤ 0.05) between control and *Tgfbr2^ΔEC^* choroids in the presence of mononuclear phagocytes (no PLX5622 treatment), but not differentially expressed between these genotypes following PLX5622-mediated MP depletion. We found 18 differentially expressed genes, with 8 of them being upregulated and 10 being downregulated in *Tgfbr2^ΔEC^* mice (**Table 1**).

**Table 1:** Differentially expressed genes exclusively regulated in control -PLX vs. *Tgfbr2^ΔEC^* -PLX mice and not regulated in PLX-treated, microglia depleted, control vs. *Tgfbr2^ΔEC^* mice.

gene	Control -PLX vs. *Tgfbr2^ΔEC^* -PLX		Control +PLX vs. *Tgfbr2^ΔEC^* +PLX	
	log2FoldChange	p. adj.	log2FoldChange	p. adj.
Fga	28, 33	5, 45E-06	0, 01	1, 00
Dpep2	18, 27	5, 26E-03	-0, 02	1, 00
Gm49354	16, 56	7, 41E-04	-0, 03	1, 00
Oprk1	16, 38	7, 66E-04	0, 00	1, 00
Zfp345	14, 50	3, 79E-03	0, 00	1, 00
Pcdha1	11, 69	6, 38E-03	0, 00	1, 00
Snhg11	0, 10	3, 96E-02	0, 00	1, 00
Rasgef1a	0, 06	2, 57E-02	0, 00	1, 00
Zfp263	-0, 02	4, 80E-02	0, 00	1, 00
Hgh1	-0, 51	5, 60E-03	0, 00	1, 00
Cdnf	-0, 60	1, 06E-02	0, 00	1, 00
Zic5	-0, 88	1, 76E-02	0, 00	1, 00
Rps18-ps5	-4, 45	3, 70E-02	0, 00	1, 00
Gm43302	-9, 08	2, 43E-02	0, 00	1, 00
Tex19.1	-17, 03	7, 31E-03	0, 02	1, 00
Pwwp4b	-17, 23	5, 15E-03	0, 06	1, 00
Vmn2r86	-21, 66	1, 74E-03	0, 01	1, 00
Gm9045	-24, 87	7, 05E-05	0, 00	1, 00

**Figure 3:**
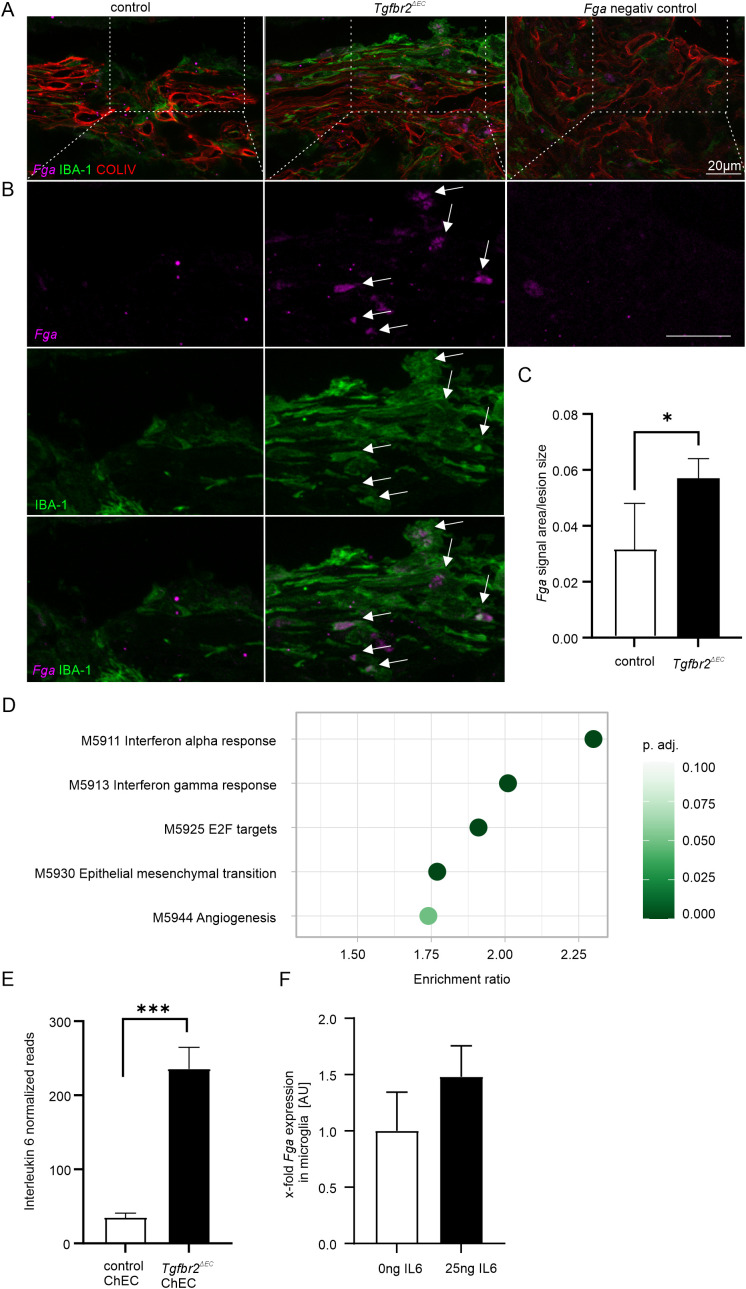
*Fga* expression in mononuclear phagocytes following laser-induced CNV in *Tgfbr2^ΔEC^* mice. **(A)** Representative overview images of CNV areas in control and *Tgfbr2^ΔEC^* mice. *Fga* mRNA is shown in magenta, IBA-1 in green, and COLIV in red. **(B)** Magnified regions from boxed areas in **(A)**. Arrows indicate colocalization of MP and Fga expression. **(C)** Quantification of *Fga*-positive area normalized to the total CNV area. (control n = 4; *Tgfbr2^ΔEC^* n = 4). Data are mean ± SEM, t-test, *p = 0.03. D and E: RNA Sequencing analysis of control ChEC and *Tgfbr2^ΔEC^*ChEC (control ChEC n = 3 *Tgfbr2^ΔEC^*ChEC n = 3). **(D)** Overrepresentation analysis (ORA) of DEGs from control ChEC in comparison to *Tgfbr2^ΔEC^*ChEC. The top 5 most significant hallmark terms are visualized. **(E)** Normalized reads for interleukin 6 in control cells and *Tgfbr2^ΔEC^*ChEC. *Adj*. p value = 2, 2E^-25^ **(F)** *Fga* mRNA expression in isolated primary microglia in response to IL6 treatment (*p* = 0.30, n= 5 per group). *** adj. p ≤ 0.001.

Following this strategy the top differentially regulated gene, Fibrinogen alpha chain (*Fga*), caught our attention, which was dramatically upregulated in the choroid of *Tgfbr2^ΔEC^* mice in the presence of MP, but completely unchanged in their absence. *Fga* is highly expressed in hepatocytes, but also in mononuclear phagocytes including microglia (https://www.proteinatlas.org/ENSG00000171560-FGA/single+cell accessed on 24^th^ Dec 2025). Notably, previous studies have implicated *Fga* in the promotion of angiogenesis (35). To confirm the induction of *Fga* expression in *Tgfbr2^ΔEC^* mice and to identify cell types that exhibit the large increase of *Fga* expression within CNV lesions, we performed RNAscope analysis. RNAscope was combined with immunohistochemical staining for IBA-1 to label MP and collagen IV to delineate CNV lesions (**Figure 3**), since we showed IBA-1 positive myeloid cells accumulated within CNV regions in the choroids of both control and *Tgfbr2^ΔEC^* mice (**Figures 2C, D**, **3A, B**).

In control mice, *Fga* signals were sparse and detected only at isolated sites. In contrast, *Tgfbr2^ΔEC^* mice exhibited markedly stronger *Fga* signals, which were significantly increased in size compared with controls (mean ± SEM: control = 0.03114 ± 0.008463, n = 4; *Tgfbr2^ΔEC^* = 0.05702 ± 0.003492, n = 4; *p* = 0.03; **Figure 3C**). Moreover, we detected a substantial proportion of these signals co-localizing with IBA-1 positive cells. In summary, the RNAscope analyses confirmed the transcriptomic findings within the lesion microenvironment and showed that MP represent the predominant source of *Fga* expression in CNV lesions in the absence of endothelial TGβ signaling (**Figures 3A, B**).

As a last step, we aimed to systematically identify the molecular mediator in this angiogenic interaction originating from Tgfbr2-deficient endothelial cells and aiming toward MP. To this end, we generated an immortalized, choroidal endothelial cell line (ChEC) from Cdh5-CreERT2; Tgfbr2fl/fl mice which were used in this study. We recently published a comprehensive characterization of immortalized choroidal endothelial cells, as well as the detailed protocol for their generation (36, 37). We induced the deletion of *Tgfbr2* in these cells *in vitro* using tamoxifen, harvested the cells and performed RNA Sequencing. Non-induced cells served as controls. This experimental design enabled a direct comparison between *Tgfbr2*-competent control ChECs and *Tgfbr2*-deficient choroidal endothelial cells (*Tgfbr2^ΔEC^* ChEC), allowing us to isolate transcriptional changes in choroidal endothelial cells driven specifically by loss of *Tgfbr2*. Using this approach, we identified a total of 3928 differentially expressed genes (DEGs) (cutoff criteria: log2fold change (log2FC) > 1 or <−1 adj. *p* < 0.05) of which 2271 were upregulated and 1657 were downregulated in *Tgfbr2^ΔEC^* ChEC (**Supplementary Table 1**). To systematically investigate to which processes these DEGs contribute to, we performed a Hallmark Gene Set Over-Representation Analysis (ORA). Among the top five significantly enriched Hallmark pathways, M5911 interferon alpha response emerged as the most significant enriched Hallmark (**Figure 3D**; **Supplementary Table 2**; adj. *p* = 9.12E^-14^). Within this enriched Hallmark gene set, we further interrogated genes previously implicated in the regulation of *Fga* expression. This targeted approach identified interleukin 6 (*Il6*) as the most promising candidate mediator (**Figure 3E**), which is supported by prior studies reporting *Il6*-dependent induction of *Fga* expression (38, 39). To test, if IL6 can induce *Fga* expression in microglia cells, we isolated primary microglia from wildtype mice and treated them with recombinant IL6 *in vitro*. qPCR analysis indeed demonstrated higher *Fga* levels in IL6 treated microglia when compared to control treated microglia (**Figure 3F**).

## Discussion

The present study demonstrates that deletion of endothelial TGFβ signaling results in more pronounced CNV lesions, an effect that is dependent on the presence of mononuclear phagocytes (MP). Depletion of MP completely rescued the increased lesion size in TGFβRII deficient mice, while not affecting CNV lesion size in TGFβRII-competent control mice, suggesting a TGFβ-dependent interaction between endothelial cells and MP. Transcriptomic and RNAscope analyses further showed that MP-derived FGA may act as a molecular mediator of this phenotype and spatial analyses confirmed that a substantial proportion of *Fga* signals co-localized with IBA-1–positive MP within CNV lesions, supporting the notion that MP represent the predominant source of FGA under conditions of endothelial TGFβ deficiency.

### Dual and context-dependent effects of TGFβ signaling in nAMD

The TGFβ signaling pathway has long been implicated as a regulator of nAMD and associated choroidal neovascularization, but its precise role remains controversial, as both activation and inhibition of TGFβ signaling have been linked to nAMD (40, 41). Numerous studies suggest that TGFβ signaling can reduce or even prevent CNV development, indicating a strong anti-angiogenic effect (24, 42–45). Supporting this concept, *in vitro* analyses showed that Müller glial cell-derived TGFβ2 inhibits proliferation of retinal endothelial cells (46). In line with this, our group showed that an endothelial cell–specific deletion of TGFβRII during retinal development leads to spontaneous CNV formation in mice, closely resembling key pathological features of human nAMD (24); an observation recently confirmed by Wang al (47). However, other studies suggest a pro-angiogenic function of TGFβ signaling, as pharmacological blockade of TGFβ signaling - via inhibitory peptides, TGFβ1-neutralizing antibodies, or TGFβRI inhibitors - reduced VEGF-A expression and attenuated CNV lesions (48–50).

Clinical data further highlight the complexity of TGFβ signaling in nAMD by showing a divergent regulation of TGFβ isoforms in nAMD: TGFβ2 levels are significantly reduced in the aqueous humor (42), whereas TGFβ1 is elevated in the vitreous, aqueous humor, and retinal pigment epithelium in nAMD patients (42–45). Experimental models are consistent with these findings, showing increased TGFβ1 and TGFβ2 expression in the retina and choroid following laser-induced CNV (43, 50, 51).

Taken together, these data underscore the dual, context-dependent role of TGFβ signaling in CNV and nAMD pathophysiology. These opposing observations regarding the function of TGFβ signaling may appear surprising at first glance. However, TGFβ-mediated stimulation or inhibition of endothelial cell proliferation represents a well-established example of how distinct receptor complexes can elicit opposing cellular responses (52, 53).

In this context, endothelial cells express two different types of TGFβ I receptors: the endothelial-specific activin receptor-like kinase (ALK)1, which phosphorylates Smad1/5/8, to promote endothelial cell motility and vascular instability (54–56), and ALK5, which phosphorylates Smad2/3 (57) and exerts anti-proliferative and vessel-stabilizing effects through downstream signaling mechanisms (52, 58). The balance between ALK1- and ALK5-mediated signaling is concentration-dependent and can determine whether TGFβ exerts pro- or anti-angiogenic effects (52, 58, 59). In this regard and based on our published data (24) and the data recently published by Wang et al. (47), it is reasonable to hypothesize that deletion of endothelial TGFβ signaling leads to a pro-angiogenic microenvironment at the interface between the choroid and RPE. However, the data obtained in this study show for the first time that this is mediated by a complex cell-cell interaction between TGFβ signaling-deficient endothelial cells and mononuclear phagocytes.

### The immune modulating role of endothelial TGFβ signaling

TGFβ signaling is well known to maintain the immune privilege of the eye (29–31). Consistent with this role, we recently demonstrated that deletion of TGFβ signaling throughout the eye results in neuroinflammation concomitant with an accumulation of myeloid cells in the retina and choroid (24, 60). Similarly, endothelial-specific deletion of TGFβ signaling in juvenile mice leads to elevated numbers of immune cells, including myeloid cells, in the retina (24, 47). In contrast, endothelial-specific deletion of *Tgfbr2* (this study) or *Tgfbr1* (Wang et al. (47)) mice at a postnatal stage beyond the initial retinal vascular expansion did not affect the myeloid cell number in the retina or choroid.

Our data demonstrate that the enlarged lesions observed in *Tgfbr2^ΔEC^* mice strictly depend on the presence of MP, as MP depletion rescued the CNV phenotype in *Tgfbr2^ΔEC^* mice without affecting lesion size in control mice. We therefore conclude that the pro-angiogenic shift responsible for increased CNV formation in *Tgfbr2^ΔEC^* mice is not caused by the deletion of TGFβRII in endothelial cells in itself.

This finding strongly suggests further that endothelial TGFβ deficiency does not act in a cell-autonomous manner, but rather requires MP-derived signals to promote pathological angiogenesis. We therefore propose a TGFβ-dependent interaction between endothelial cells and MP in the regulation of CNV development. During previous fate-mapping studies in laser-induced CNV and other ocular neovascularization models we consistently demonstrated that resident microglia represent the predominant myeloid population at neovascular sites including CNV lesions, whereas the contribution of blood-derived monocytes/macrophages is minimal (61–63). Therefore, we strongly assume that the effects mediated by PLX are primarily attributable to microglial depletion, although a contribution of infiltrating macrophages cannot be entirely excluded.

Adding to the proposed TGFβ-dependent endothelial microglial interaction, we identified the MP-derived molecular factor *Fga* contributing to this. As our RNA sequencing analysis was performed on lesion-enriched tissue containing heterogeneous cell populations, a contribution of subtle differences in cellular composition cannot be fully excluded. We therefore performed quantitative and spatial analyses on CNV lesions that strongly support a MP–specific upregulation of *Fga* rather than a predominant effect of altered cell abundance. FGA is the alpha component of fibrinogen (64), traditionally regarded as a hepatocyte-derived coagulation factor. However, accumulating evidence indicates that fibrinogen chains, such as FGA can be locally expressed by myeloid cells and have potent angiogenic effects (65) (https://www.proteinatlas.org/ENSG00000171560-FGA/single+cell accessed on 24^th^ December 2025).

In this context, FGA can regulate the expression of both VEGF and VEGFR2, thereby promoting angiogenesis (35). As a component of the extracellular matrix (ECM), FGA also provides a scaffold that supports endothelial cell adhesion, facilitating key aspects of neovascularization and vessel stabilization (35, 66, 67). Furthermore, fibrin can bind to CD11c, thereby activating CD11c^+^ immune cells (68). CD11c^+^ myeloid cells have been identified as pro-angiogenic effectors in laser-induced CNV models (69, 70), suggesting that fibrin(ogen)-mediated activation of CD11c^+^ cells may further amplify angiogenic responses. Additionally, *Fga* was previously detected as a component of drusen, providing early evidence for its potential role in age-related macular degeneration (71). Furthermore, fibrinogen has been shown to increase VEGF synthesis in RPE cells and choroidal endothelial cells, thereby stimulating angiogenesis and likely contributing to CNV formation (72, 73). Although cell culture experiments using a hepatocyte cell line demonstrated that TGFβ can suppress *Fga* expression within a single cell type (74), the mechanisms governing this regulation across distinct cell types remain to be fully elucidated. Our findings demonstrate, that deletion of TGβ signaling in choroidal endothelial cells results in a strong upregulation of interleukin 6 (*Il6*), a cytokine known to induce *Fga* expression (38, 39). This observation suggests that IL6 may contribute to the proposed endothelial-MP/microglial signaling axis underlying *Fga* induction. While IL6 treatment of microglia was associated with a trend toward increased *Fga* expression, additional studies will be required to further validate the exact contribution of this pathway. Still, our findings clearly underscore the functional relevance of FGA in mediating angiogenic interaction between endothelial cells and mononuclear phagocytes, such as microglia.

## Conclusion

Our study reveals a critical interplay between endothelial TGFβ signaling and mononuclear phagocytes, such as microglia in the regulation of CNV. Endothelial TGFβRII deficiency alone is not sufficient to exacerbate CNV; rather, MP are required to mediate this effect, with FGA possibly acting as a key molecular mediator. These findings suggest a novel endothelial–microglia interaction that contributes to pathological angiogenesis and oBRB dysfunction in nAMD.

## Materials and methods

### Mice

All procedures conformed to the tenets of the National Institutes of Health Guidelines on the Care and Use of Animals in Research, the EU Directive, 2010/63/E and institutional guidelines. The mice were on a C57BL/6J background and kept in a 12 h light/dark cycle. Mice with two floxed alleles for *Tgfbr2* (*Tgfbr2^fl/fl^*) were crossed with animals that heterozygously expressed a *Cdh5*-controlled, tamoxifen-inducible Cre recombinase (*Cdh5-Cre^ERT2^*) resulting in *Tgfbr2^fl/fl^; Cdh5-Cre^ERT2^* and *Tgfbr2^fl/fl^* mice (75, 76). Both sexes were used for experiments. The efficiency of the Cre recombinase activation in *Cdh5-Cre^ERT2^* mice was confirmed using *mT/mG* reporter mice (77). All animal experiments were authorized by the local animal care and use committee under the respective EU, national, federal and institutional regulations for animal experiments (ethical protocol number AZ 55.2.2-2532-2-956).

### Tamoxifen treatment

For activation of the *Cdh5*-Cre-ERT2 recombinase activity in adult animals, 6- to 7-week-old *Cdh5-Cre^ERT2^; Tgfbr2^fl/fl^* mice (*Tgfbr2^ΔEC^*) and *Tgfbr2^fl/fl^* (control) littermates of mixed gender were treated with Tamoxifen (TAM, 13258, Cayman Chemical Company, Ann Arbor, MI, USA). To this end Tamoxifen was dissolved in corn oil (Sigma-Aldrich, C8267) and TAM eye drops (20 mg/ml) were applied topically by pipette (10 µl/drop) onto each mouse eye times per day for five consecutive days. Successful deletion of Tgfbr2 was confirmed using PCR (5′-TAAACAAGGTCCGGAGCCCA-3′ (sense) and 5′AGAGTGAAGCCGTGGTAGGTGAGCTTG-3′ (antisense)) and qPCR (5′-AGAAGCCGCATGAAGTCTG-3′(sense) and 5′-GGCAAACCGTCTCCAGAGTA-3 (antisense)) of choroidal tissue as described in (75, 78).

### Depletion of mononuclear phagocytes

To induce MP depletion in a subset of *Tgfbr2^ΔEC^* and *Tgfbr2^fl/fl^* mice, a PLX5622 (Plexxikon Inc., Berkeley, California, USA) containing diet was fed starting at the time of the first tamoxifen application until the end of the experiment. Another subset of *Tgfbr2^ΔEC^* and *Tgfbr2^fl/fl^* mice received control food and therefore did not harbor microglia cell depletion.

### Laser-induced CNV and *in vivo* imaging

Anesthetized mice with dilated pupils were placed in front of a frequency doubled NdYAG laser (IRIDEX OcuLight TX Green 532 nm) Laser burns at equal distance from the optic disc were applied using a power of 150 mW, a fixed diameter of 100 µm, and a duration of 100 ms. For immunohistochemical analyses three laser burns per eye were applied. For molecular analyses (RNA sequencing) six laser burns per eye were applied to maximize the enrichment with diseased tissue. Only the central part of the choroidal tissue (with the laser lesions) was used for the RNA sequencing analyses. After laser treatment color fundus photography (CFP, Micron IV camera and Micron Discover software) was performed. Mice were sacrificed at day (d) 3 for RNA sequencing and at d7 after performing CFP and fluorescence angiography (Fluorescein (Alcon 10%) was diluted 1: 20 with 0.9% NaCl and 10 µL/g mouse bodyweight were injected) for immunohistochemistry. Animals with subretinal bleedings or confluent lesions were excluded from further analysis.

### Flatmount staining and microscopy

For fluorescence microscopy, mice were perfused with 10 ml PBS followed by 10 ml 4% paraformaldehyde (PFA). After enucleation at d7, paraformaldehyde (PFA) -fixed eye cups were dissected to perform a collagen IV (COL IV) and ionized calcium-binding adapter molecule 1 (IBA 1). After blocking, primary antibodies against IBA 1 (019-19741, Wako, Neuss, Germany) and COL IV (AB769, Merck Millipore, Billerica, MA, USA) were added over two nights at a dilution of 1:500 at 4 °C. Afterwards, secondary antibodies were added at a dilution of 1:500 (Alexa Fluor^®^ 568 and Alexa Flour^®^ 488, Life Technologies, Carlsbad, CA, USA) overnight at 4 °C in the dark. Using the Axio Imager.M2 fluorescence microscope (Zeiss, Jena, Germany), the Axiocam 506 camera, and the corresponding Axiovision software version, choroidal overview images were taken (Plan-Apochromat 10x/0.3). ZEN 3.5 software (blue edition, Zeiss, Jena. Germany) was used to measure the CNV area. The number of IBA1-positive microglial cells in the immediate vicinity of the laser-induced CNV area was manually quantified using the multipoint tool (ImageJ 1.53k software, Wayne Rasband, National Institutes of Health, Bethesda, MD, USA).

### Paraffin embedding and RNA scope

Prior to RNAscope, eyes were fixed for 4 hours in 4% PFA, washed extensively in PBS, and embedded in paraffin, according to standard protocols. Paraffin sections (6 μm thick) stretching through CNV lesions were deparaffinized and permeabilized according to the multiplex fluorescence detection kit v2 user manual by ACD using ACD HybEZ™ II hybridization system. Target retrieval was performed at 99 °C for 15 minutes. Slides were then washed in a. dest., and 100% ethanol. After drying at 60 °C for 5 minutes RNAscope^®^ protocol was continued following the manufacturer`s instructions. For hybridization Mm-*Fga*-C3 probe (biotechne, 578911-C3) was used. After amplification, the probe was detected using TSA (tyramide signal amplification) vivid 570. Slides were immunohistochemically stained immediately after RNAscope^®^ with antibodies against IBA-1 (abcam 178846) and COLIV (bio-rad 134001) using standard protocols (79).

Slides were counter stained with DAPI and mounted using ProLong Gold Antifade Mountain solution. Lastly, samples were examined via ZEISS Axio Observer with ZEISS ApoTome 2 and quantified using ZEISS Zen 3.0 imaging processing software. Total *Fga* signal areas were determined using Zeiss Zen 3.0 imaging processing software (Zeiss, Jena, Germany) and normalized to CNV area.

### Microglia isolation, IL6 treatment and *Fga* mRNA expression

Microglia were isolated from the cortex of C57BL/6J mouse pubs. Briefly: the cortex were isolated, meninges removed to prevent the contamination with other immune cells and the tissue was treated with trypsin/EDTA (0.05%/0.02%, Sigma Aldrich) at 37 °C. After homogenization of tissue and filtration via cell strainer (70 µm) the cell suspension was centrifuged, resuspended in 5 ml DMEM with 15% FCS and 15 µg/ml gentamicin. Cells were seeded on poly-D-lysin coated cell culture flasks and incubated at 37 °C, 5% CO_2_ for 3 days. Interleukin-6 (IL-6, PeproTech 216-16-50UG) treatment was performed as soon as cells were confluent. Cells were treated first with 25 ng/ml for 24h at 37 °C, 5% CO_2_ and microglia were harvested by shaking flasks for 2h at 220 rpm. Supernatants were then centrifuged for 8 minutes at 5000 rpm, 4 °C and cell pellets were used for RNA Isolation. RNA was isolated using Qiagen RNeasy Plus Micro Kit (74034), followed by cDNA synthesis (Eurogentec Reverse Transcriptase Core Kit RT-RTCK-03) and qPCR (Taykon B0701) as described by manufacturer. *Fga* primers were: fw: GCCCAAGAGTTGTGGAGAGA rev: AAGGGCATTTGTGGTTCCAGT, *Rpl32* was used as a housekeeper.

### Generation of immortalized choroidal endothelial cells

Choroidal tissue was isolated from *Cdh5-Cre^ERT2^; Tgfbr2^fl/fl^* mice and used for the generation of an immortalized choroidal endothelial cell (ChEC) line according to previously published protocols (36, 37). For induction of Cre-mediated recombination, ChEC were treated with 100 nM 4-hydroxytamoxifen (4-OHT, Sigma-Aldrich, Germany) for 72 h (referred to as *Tgfbr2^ΔEC^*ChEC), which resulted in a significant reduction of *Tgfbr2*/TGFBR2 expression. Non-tamoxifen-treated cells from the same cell line were used as controls (control ChEC).

### RNA extraction for RNA sequencing

Total RNA was extracted from mouse choroid tissue stabilized in RNAlater buffer according to the “Purification of total RNA from animal and human tissue” protocol of the RNeasy Micro Kit (QIAGEN, Hilden, Germany).

In brief, the tissue sample was stored in RNAlater and shipped at 2-8 °C. After centrifuging for 5 minutes at 5, 000 x g, the RNAlater was removed and the sample was disrupted and homogenized in 350 µl RLT buffer containing 1% beta-mercaptoethanol with Precellys CK14 ceramic beads (1 cycle of 15 seconds at 5500 rpm) using a Precellys 24 Homogenisator (Bertin Corp., Rockville, MD, USA). Next the sample was centrifuged for 2 min at full speed and 350 µl of the cleared supernatant was transferred to a new tube. One volume of 70% ethanol was added and the sample was applied to a RNeasy MinElute spin column followed by an on-column DNase digestion and several wash steps. Finally total RNA was eluted in 14 μl of nuclease free water. Purity and integrity of the RNA was assessed on the Agilent 2100 Bioanalyzer with the RNA 6000 Nano LabChip reagent set (Agilent, Palo Alto, CA, USA). For RNA Sequencing of immortalized cells, cells were harvested and total RNA was extracted using TRIzol reagent according to the manufacturer’s instructions.

### RNA sequencing

Library preparation and RNAseq of mouse choroidal tissue were carried out as described in the Illumina “Stranded mRNA Prep Ligation” Reference Guide, the Illumina NextSeq 2000 Sequencing System Guide (Illumina, Inc., San Diego, CA, USA), and the KAPA Library Quantification Kit - Illumina/ABI Prism (Roche Sequencing Solutions, Inc., Pleasanton, CA, USA). In brief, 200 ng of total RNA was used for purifying the poly-A containing mRNA molecules using oligo(dT) magnetic beads. Following purification, the mRNA was fragmented to an average insert size of 200–400 bases using divalent cations under elevated temperature (94 °C for 8 minutes). Next, the cleaved RNA fragments were reverse transcribed into first strand complementary DNA (cDNA) using reverse transcriptase and random hexamer primers. Thereby Actinomycin D was added to allow RNA-dependent synthesis and to improve strand specificity by preventing spurious DNA-dependent synthesis. Blunt-ended second strand cDNA was synthesized using DNA Polymerase I, RNase H and dUTP nucleotides. The incorporation of dUTP, in place of dTTP, quenches the second strand during the later PCR amplification, because the polymerase does not incorporate past this nucleotide. The resulting cDNA fragments were adenylated at the 3’ ends and the pre-index anchors were ligated. Finally DNA libraries were created were created using a 13 cycles PCR to selectively amplify the anchor-ligated DNA fragments and to add the unique dual indexing (i7 and I5) adapters. The libraries were bead purified twice and quantified using the KAPA Library Quantification Kit. Equimolar amounts of each library were sequenced on an Illumina NextSeq 2000 instrument controlled by the NextSeq 2000 Control Software (NCS) v1.5.0.42699, using one 50 cycles P3 Flow Cell with the dual index, single-read (SR) run parameters. Image analysis and base calling were done by the Real Time Analysis Software (RTA) v3.10.30. The resulting.cbcl files were converted into.fastq files with the bcl2fastq v2.20 software. RNA extraction, library preparation and RNAseq of mouse choroidal tissue were performed at the Genomics Core Facility “KFB - Center of Excellence for Fluorescent Bioanalytics” (University of Regensburg, Regensburg, Germany; www.kfb-regensburg.de).

For immortalized cell lines, strand-specific transcriptome libraries were prepared by BGI Center (Shenzhen, China) using a DNBSEQ high-throughput sequencing platform. Briefly, mRNA was purified from total RNA using oligo(dT)-coupled magnetic beads and subsequently fragmented into short. First-strand cDNA synthesis was performed using random primers, followed by second-strand cDNA synthesis incorporating dUTP instead of dTTP to preserve strand specificity. Double-stranded cDNA was purified using magnetic beads. Following cDNA synthesis, end repair and adenylation of the 3′ ends were performed prior to adaptor ligation. Ligated products were purified and subjected to PCR amplification after digestion of the dUTP-containing second strand using uracil-DNA glycosylase (UDG). Amplified libraries were purified using XP beads and validated on an Agilent 2100 Bioanalyzer. For sequencing, purified PCR products were heat-denatured and circularized using splint oligonucleotides to generate single-stranded circular DNA libraries. DNA nanoballs (DNBs) were subsequently generated by phi29-mediated rolling circle amplification and loaded onto patterned nanoarrays. Sequencing was performed on the DNBSEQ platform.

### Bioinformatics

FastQ files were quality checked with FastQC v0.11.5 (80). All files passed QC. The reads were pseudo-aligned against Ensembl *mus musculus* GRCm39 release vM29 and quantified using salmon v1.10.1 (81) using a mean insert size of 310 ± 80 bp as determined by Bioanalyzer (Agilent) traces for each library. Datasets for retinal and choroidal samples were separately analyzed from here on. Samples were screened for outliers using a combined PCA and clustering analysis. To this end, counts were imported using tximport v1.24.0 (82) into DESeq2 v1.36.0 (83) and an intercept matrix of all samples was computed. This matrix was corrected for sex of the mice using limma v3.52.4 (84). A sample was defined as an outlier if it was outside a 68% probability ellipse in PCA analysis (85) and was outside a -2.5 standardized connectivity cutoff of an Euclidean distance matrix of all samples (86). No outlier samples were identified for the choroidal dataset. We next computed TPMs and used the xCell (87) website (https://comphealth.ucsf.edu/app/xcell) to approximate the percentage of neuronal cells in each sample. The neuronal percentage as computed by xCell was between 0 and 6.9% for the choroidal dataset. Transcriptional dysregulation was computed as above with the exception of using a design matrix that included sex of the mice and the percentage of neuronal cells as covariates and genotype, as well as treatment as the variables of interest. Ashr was used as the fold change shrinkage estimator (88). Scripts are available upon request. For RNA Seq data of immortalized choroidal endothelial cells, reads were processed with fastp v0.23.2 (89) to remove sequences originating from sequencing adapters and sequences of low quality using the program’s default parameters and the --correction option. Reads were then aligned to the murine reference assembly GRCm38.110 using STAR v2.7.10a (90). Differential expression was assessed using DESeq2 v1.42.0 (83). A gene was considered significantly differentially expressed if the corresponding absolute log2-transformed fold change (log2FC) was not less than 1 and, in addition, the Benjamini–Hochberg adjusted p-value did not exceed a value of 0.05. Overrepresentation (ORA) was performed with clusterProfiler v4.10 (91) in combination with Molecular Signatures Database (MSigDB) Hallmark gene sets (92).

### Statistical analysis

Statistical analysis was performed using GraphPad Prism (GraphPad Software, Version 6.0, La Jolla, CA, USA). Data was tested for normal distribution. Two-way ANOVA analyses were performed if more than two individual groups were compared (*post-hoc* test: Tukey). A Student`s t-test was performed to compare the mean variables of two individual test populations. Differences were considered significant when *p*-value was < 0.05.

## Data Availability

The datasets presented in this study can be found in online repositories. The names of the repository/repositories and accession number(s) can be found below: https://www.ncbi.nlm.nih.gov/geo/, GSE318434.

## Supplementary material

Supplementary Figure 1 Successful deletion of Tgfbr2 in choroidal endothelium. **(A)***, Tgfbr2* deletion PCR with choroidal genomic DNA of an adult *Tgfbr2*^Δ^*EC* mouse and its control littermate. Due to their different product sizes (product size control: 3974 bp, product size after Tgfbr2 deletion: 636 bp) and the PCR elongation time of 1 min, the Tgfbr2 deletion PCR product was only amplified after Cre-mediated recombination. **(B)**. Real-time RT-PCR in adult control and *Tgfbr2^EC^* choroideae. Mean ± SEM control (n = 6) = 1.000 ± 0.05490; *Tgfbr2^ΔEC^* (n = 7) = 0.6761 ± 0.07106; *p* = 0.0049. **(C).** Representative images of *Cdh5^CreERT2^;mT/mG* and Cre negative *mT/mG* control reporter mice demonstrate successful and endothelial-specific activation of Cre recombinase following tamoxifen treatment. Upper left image shows a cryosection of an entire eye illustrating Cre activation in the endothelium of the iris (I), retina (R), and choroid (C). The right column presents retinal flatmounts, including low-magnification overviews (top and bottom panels) and a higher-magnification inset (middle panel). Left column visualizes cyro sections through the retina and the adjacent choroid (GCL: ganglion cell layer; IPL: inner plexiforme layer; OPL: outer plexiforme layer; C: choroid).

Supplementary Figure 2 Successful depletion of microglia cells in the retinae of control and *Tgfbr2^EC^* following PLX5622 treatment. **(A)** Representative images of IBA-1 staining in retinae untreated retinae (d0) and of PLX5622 treated mice at two (d2), seven (d7) and fourteen (d14) days of treatment. Only a few IBA-1-positive cells were detectable at d2, as shown by the cell circled in white. B and C. Quantification of IBA-1 positive cells in peripheral **(B)** and central **(C)** retinae. d0 n= 6; d2 -PLX n= 10, +PLX n= 10; d7 -PLX n= 5, +PLX n= 5; d14 -PLX n= 4, +PLX n= 5. Data are mean ± SEM, student`s t-test, *** p ≤ 0.001.
