# Supplementary figures and images for: Endothelial TGFβ signaling modulates choroidal neovascularization severity via myeloid–endothelial cell interaction
Source: Front Immunol. 2026 Jun 26;17:1813788. doi: 10.3389/fimmu.2026.1813788 (PMC13349815; doi:10.3389/fimmu.2026.1813788)

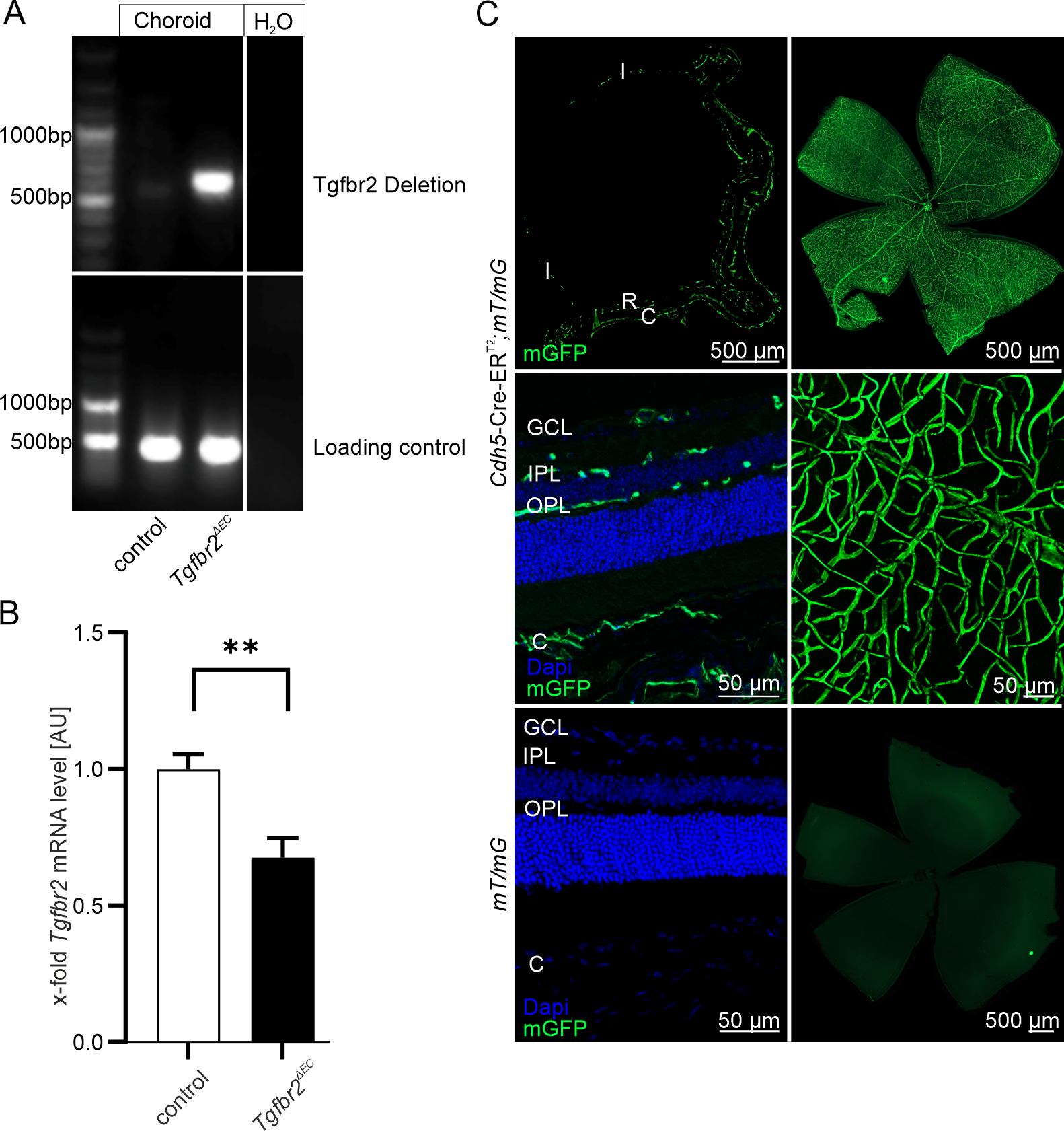

Supplement: Supplementary Figure 1 — Successful deletion of Tgfbr2 in choroidal endothelium. (A), Tgfbr2 deletion PCR with choroidal genomic DNA of an adult Tgfbr2ΔEC mouse and its control littermate. Due to their different product sizes (product size control: 3974 bp, product size after Tgfbr2 deletion: 636 bp) and the PCR elongation time of 1 min, the Tgfbr2 deletion PCR product was only amplified after Cre-mediated recombination. (B). Real-time RT-PCR in adult control and Tgfbr2EC choroideae. Mean ± SEM control (n = 6) = 1.000 ± 0.05490; Tgfbr2ΔEC (n = 7) = 0.6761 ± 0.07106; p = 0.0049. (C). Representative images of Cdh5CreERT2;mT/mG and Cre negative mT/mG control reporter mice demonstrate successful and endothelial-specific activation of Cre recombinase following tamoxifen treatment. Upper left image shows a cryosection of an entire eye illustrating Cre activation in the endothelium of the iris (I), retina (R), and choroid (C). The right column presents retinal flatmounts, including low-magnification overviews (top and bottom panels) and a higher-magnification inset (middle panel). Left column visualizes cyro sections through the retina and the adjacent choroid (GCL: ganglion cell layer; IPL: inner plexiforme layer; OPL: outer plexiforme layer; C: choroid). [file Image1.tif]

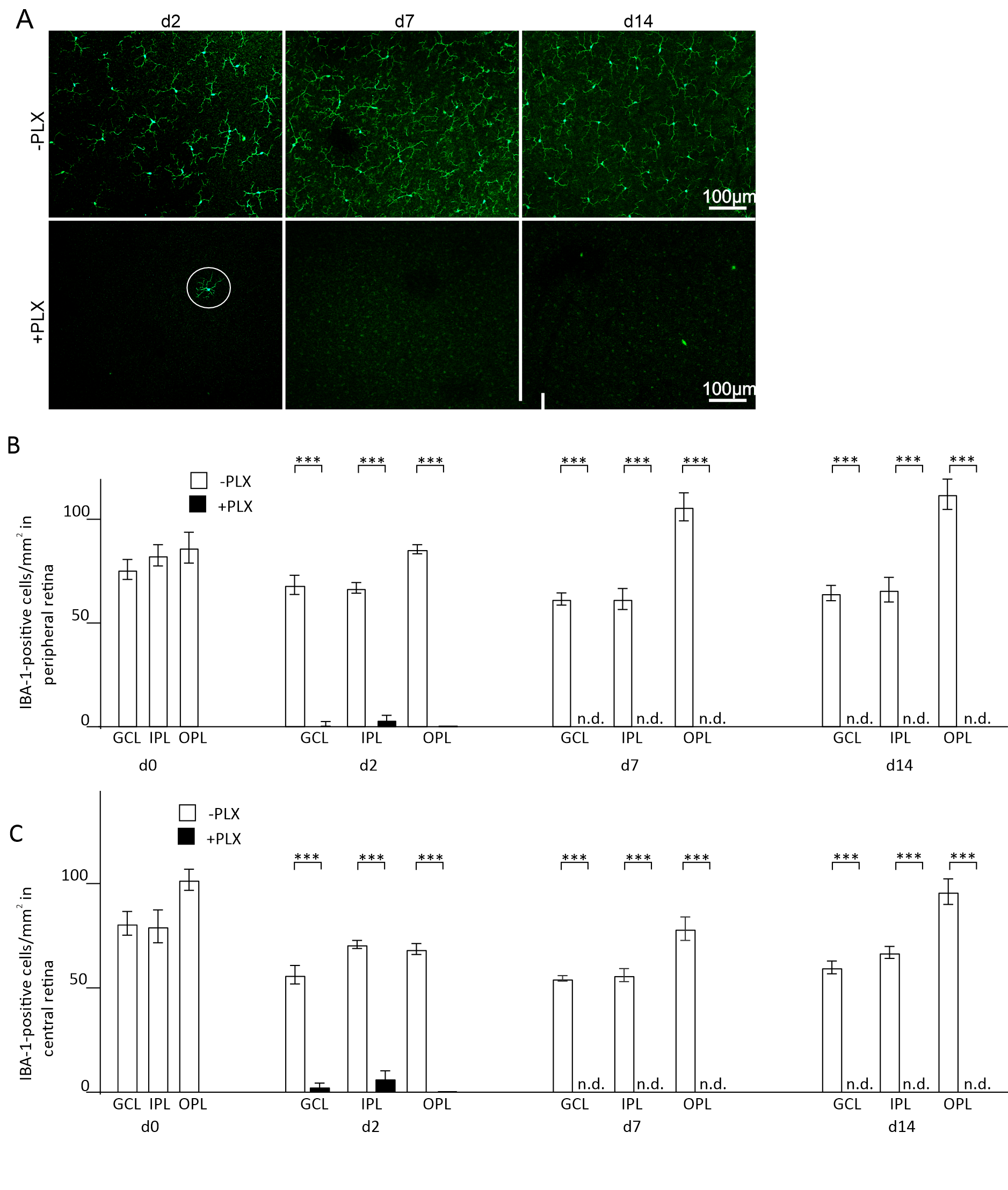

Supplement: Supplementary Figure 2 — Successful depletion of microglia cells in the retinae of control and Tgfbr2EC following PLX5622 treatment. (A) Representative images of IBA-1 staining in retinae untreated retinae (d0) and of PLX5622 treated mice at two (d2), seven (d7) and fourteen (d14) days of treatment. Only a few IBA-1-positive cells were detectable at d2, as shown by the cell circled in white. B and C. Quantification of IBA-1 positive cells in peripheral (B) and central (C) retinae. d0 n= 6; d2 -PLX n= 10, +PLX n= 10; d7 -PLX n= 5, +PLX n= 5; d14 -PLX n= 4, +PLX n= 5. Data are mean ± SEM, student`s t-test, *** p ≤ 0.001. [file Image2.tif]
